# A Case Report of Transient Amnesia Following Spontaneous Intracerebral Hemorrhage of the Fornix

**DOI:** 10.7759/cureus.34519

**Published:** 2023-02-01

**Authors:** Ali I Abu-Alya, Vishnu Halthore, Tanvir Khosla, Devraj Chavda, Venkatachalam Veerappan

**Affiliations:** 1 Neurology, Hospital Corporation of America (HCA) Healthcare, Southern Hills Hospital, Las Vegas, USA; 2 Epilepsy, Columbia University, New York City, USA; 3 Pediatric Neurology, State University of New York Downstate Medical Center, Brooklyn, USA

**Keywords:** indirect brain concussion, spontaneous intracranial hemorrhage, transient global amnesia, memory, papez circuit, fornix, intracranial hemorrhage, transient amnesia

## Abstract

In this report, we share the case of a 65-year-old male with a remote history of brain concussion who presented to the emergency department for evaluation of transient amnesia that lasted 30 minutes to one hour. He was found to have spontaneous intracerebral hemorrhage of the fornix as a cause of his amnesic episode. To date from the creation of this case report (January 2023), spontaneous hemorrhage of the fornix resulting in transient amnesia has not been previously described in the literature. The fornix is an unusual location for spontaneous hemorrhage to occur. The differential diagnosis of transient amnesia is broad and includes, but is not limited to, transient global amnesia, traumatic injury, hippocampal infraction, and various metabolic derangements. Determination of the etiology of transient amnesia can result in changes in treatment decisions. Because of this unique patient presentation, we propose that spontaneous hemorrhage of the fornix should be considered in patients who present with transient amnesia.

## Introduction

The fornix is a white matter tract that is the major output tract from the hippocampus to the mammillary bodies [1]. This is part of the Papez circuit, which plays an important role in the formation of memories [1]. Case reports exist that demonstrate amnesia following an infraction or traumatic injury to the fornix. However, no cases of spontaneous forniceal hemorrhage resulting in transient amnesia have been published in the literature [1,2]. This case is unique because it changed the way we approached a patient who presented with the chief complaint of memory loss. We found that the possible mechanisms of isolated spontaneous forniceal hemorrhage in this patient include hypertensive hemorrhage, hemorrhagic conversion of ischemic stroke, and concussive hemorrhage. Identification of the etiology of transient amnesia is important because it alters treatment decisions. In this case, we recommended against the use of antiplatelet or anticoagulant medications until the resolution of the patient’s intracranial hemorrhage. This is not a typical recommendation for patients presenting with the chief complaint of transient amnesia. This patient case adds to the literature and illustrates that it is important to evaluate for forniceal hemorrhage as a cause of transient amnesia.

## Case presentation

A 65-year-old male with a history of hypertension, obesity, and remote concussion was brought to Southern Hills Hospital emergency department in Las Vegas, Nevada, for amnesia after returning from the gym. His wife reported that he was confused and walking back and forth. He repeatedly asked questions about his schedule. His wife checked his blood pressure, which was noted to be 199/110 mmHg. His symptoms resolved for an estimated 30-60 minutes and prior to his arrival at the emergency department.

The patient recalls driving home and his onward journey to the hospital. He has no memory of what occurred in his home. He denied any recent head trauma, prior episodes of confusion, memory loss, seizures, or syncope. In the emergency department, he was found to have a blood pressure reading of 180/97 mmHg and was tachycardic (heart rate (HR): 116 bpm). His body mass index (BMI) was 34.9 kg/m^2^. On physical examination, he was alert and oriented. His short- and long-term memory was intact, and his neurological examination was unremarkable. Laboratory evaluation revealed normal white blood count, hemoglobin, hematocrit, platelet, serum sodium, serum creatinine, serum glucose, and thyroid-stimulating hormone (TSH). His urine drug screen was negative, and his alcohol level was less than 0.010%.

**Figure 1:**
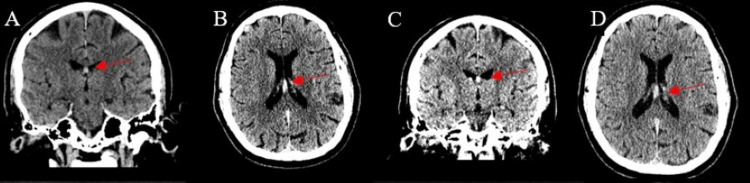
Non-contrast head CT performed during hospitalization A: Initial non-contrast head CT coronal slice demonstrating fornix hyperdensity (arrow). B: Initial non-contrast head CT axial slice with bilateral fornix hyperdensity (arrow). C: Non-contrast CT coronal slice (24 hours) unchanged fornix hyperdensity (arrow). D: Head CT axial slice (24 hours) with stable fornix hyperdensity (arrow). CT: computed tomography

**Figure 2:**
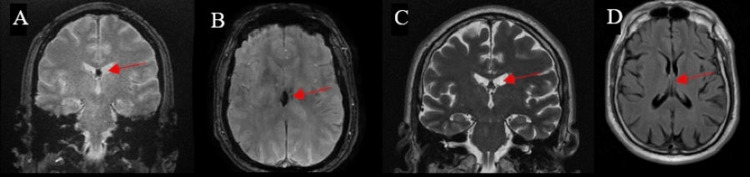
Brain MRI during hospitalization A: Brain MRI GRE coronal slice with fornix hypointensity (arrow). B: MRI GRE axial slice with fornix hypointensity (arrow). C: Non-contrast MRI T2 coronal slice with fornix hypointensity (arrow). C: Contrasted MRI T2 FLAIR axial slice of the fornix demonstrating no signs of mass effect or edema (arrow). MRI: magnetic resonance imaging, GRE: gradient echo sequence, FLAIR: fluid-attenuated inversion recovery

Head computed tomography (CT) demonstrated a small hyperdense lesion of 50 HU involving the columns of the fornix (Figure 1A-1B). Brain magnetic resonance imaging (MRI) demonstrated a 5.5 × 8.5 × 7.5-mm region of low gradient echo signal in the posterior fornix (Figure 2A-2D). The signal abnormality lacked mass effect, edema, or diffusion signal abnormality. Based on the imaging findings, the differential included hemorrhage or calcification. Because of the lack of mass effect or edema, initially, intracranial calcification was favored as an etiology over acute hemorrhage. Brain magnetic resonance angiography (MRA) was performed and was unremarkable. Electroencephalogram (EEG) demonstrated no epileptiform abnormalities. The following day, head CT demonstrated a stable hyperdensity of the fornix (Figure 1C-1D). He remained in the hospital overnight for observation. His blood pressure improved without treatment, and he did not demonstrate any neurological deficits during his hospital stay. He was then discharged home.

**Figure 3:**
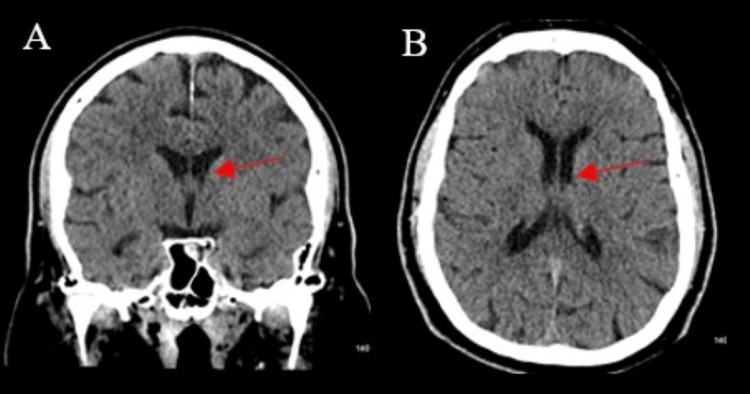
Brain CT at two-month follow-up A: Two-month non-contrast head CT coronal slice demonstrating no hyperdense lesion of the fornix (arrow). B: Two-month non-contrast head CT axial slice demonstrating no hyperdense lesion of the fornix (arrow). CT: computed tomography

At follow-up two and four months after hospitalization, he did not have any further episodes of amnesia or memory problems. His bedside memory testing did not demonstrate any cognitive issues. A repeat head CT demonstrated the resolution of the hemorrhage (Figure 3A-3B). He underwent a repeat EEG, which was negative for any epileptiform abnormalities.

## Discussion

This patient’s presentation is unusual as spontaneous hemorrhage of the fornix is atypical. The differential diagnoses we initially thought that could be a cause of his symptoms included transient global amnesia, temporal lobe epilepsy, metabolic encephalopathy, ischemia of the hippocampus, and posterior reversible encephalopathy syndrome. Spontaneous forniceal hemorrhage is not a known etiology of transient amnesia but was discovered as a cause of his symptoms after thorough evaluation and investigations were performed. The likely cause of amnesia due to forniceal hemorrhage is the disruption of the Papez circuit. Despite incurring a forniceal hemorrhage, the patient exhibited no obvious memory deficits by the time he presented to the emergency room as well as on follow-up evaluations.

In the Intensive Blood Pressure Reduction in Acute Cerebral Hemorrhage Trial (INTERACT2) study, data from over 2,000 patients with image-proven intracerebral hemorrhage was assessed [3]. Secondary analysis of the data has been performed to determine intracerebral hemorrhage location and its relationship to outcome [4]. None of the hemorrhage patterns found in the secondary analysis included the fornix alone.

Forniceal columns receive their vascular supply from short branches of the anterior communicating artery [5]. Given the findings of poorly controlled hypertension, his intracerebral hemorrhage could conceivably be caused by lipohyalinosis of these short branches secondary to chronic hypertension, followed by arterial rupture around the time of the amnesic episode. Alternatively, the patient could have incurred an ischemic infarct of the fornix that underwent hemorrhagic conversion by the time the patient presented to the emergency room, although this is less likely as no areas of restricted diffusion were found on MRI diffusion-weighted imaging (DWI).

Another potential mechanism of the patient’s spontaneous forniceal hemorrhage may have been related to his activities at the gym. As reported throughout concussion literature, rapid acceleration and deceleration forces on the brain are thought to result in stretching and potential disruption of all of its components, including its vasculature [6]. Although he denied head trauma, he may have made sudden movements of his head during his exercise routine, causing indirect concussion with rupture of the small vessels feeding the fornix.

The patient was assessed at multiple follow-up office appointments by two separate neurology practitioners and found to have no obvious clinically relevant memory deficits. Due to the importance of the fornix in memory formation, perhaps the patient would show some deficits in memory if he were to have undergone in-depth pre- and post-insult neuropsychological testing.

## Conclusions

Being the output tract of the hippocampus and part of the Papez circuit, the fornix is important in the formation of memory. As such, lesions to the fornix can result in an amnesic episode. Although rare, spontaneous hemorrhage of the fornix should be considered in the differential diagnosis of patients presenting with transient amnesia. This case is unique because it changed the way we approached a patient who presented with the chief complaint of memory loss and resulted in a change in management from the typical patient presenting with transient memory loss. This patient case adds to the literature and illustrates that it is important to evaluate for forniceal hemorrhage as a cause of transient amnesia. Potential mechanisms of isolated spontaneous hemorrhage of the fornix include hypertension, ischemic stroke with hemorrhagic conversion, or indirect brain injury. More clinical observations and research in the etiology and long-term clinical course of patients presenting with spontaneous fornix hemorrhage would be informative and impact clinical management.
